# A study examining whether social cognitive abilities impact on recovery from PTSD

**DOI:** 10.1192/bjo.2021.798

**Published:** 2021-06-18

**Authors:** Chantelle Wiseman, Jonathan Bisson, Anke Karl, Andrew Lawrence, James Hotham, Stan Zammit

**Affiliations:** 1 University of Bristol; 2 Cardiff University; 3 Exeter University; 4 Avon and Wiltshire Partnership Trust; 5 Cardiff University, Exeter University

## Abstract

**Aims:**

Deficits in social cognition (the ability to recognise and understand emotions, intentions and actions in oneself and in others) have been found in people with post-traumatic stress disorder (PTSD). Few studies so far have examined whether social cognitive ability impacts on PTSD recovery. Here we present a protocol and preliminary data for a study that aims to evaluate whether pre-treatment social cognitive deficits are associated with treatment outcomes following trauma-focused therapy for PTSD.

**Method:**

The protocol was developed after discussion with Patient and Public Involvement (PPI) groups, and a battery of social cognitive tasks was trialled on 20 participants without PTSD. The final protocol was then developed using information and feedback from these preliminary studies. We aim to recruit 60 individuals who are about to start a trauma-focused therapy for PTSD within the two tertiary PTSD services. Social cognition (measured using a variety of tasks including Reading the Mind in the Eyes Task and the Reflective Functioning Questionnaire) and potential confounders (including severity of trauma history, attachment and verbal IQ) are assessed at baseline, prior to the start of therapy. PTSD symptom severity (measured using the PCL-5) and daily functioning (measured using the WSAS) are assessed pre and post-treatment. The primary aim of the study is to examine whether baseline social cognition is associated with the degree of improvement in the PCL-5.

**Result:**

So far 29 participants have been recruited and consented. 6 participants have completed follow-up assessments. The study has been adapted for the COVID-19 pandemic so participants can complete the tasks remotely. Preliminary results show a moderate negative correlation between baseline social cognitive abilities and baseline PTSD symptom severity (Spearman's correlation -0.30) and functional abilities (Spearman's correlation -0.42).

**Conclusion:**

Development of our study in collaboration with PPI and preliminary testing of measures suggests it is likely that it will be feasible for us to conduct this study in this patient group. Baseline preliminary results show/suggest a moderate correlation between PTSD symptom severity and social cognitive impairment. Our main analyses, when completed, will help to determine whether social cognitive ability is associated with recovery from PTSD.

